# Effectiveness of naloxone distribution in community settings to reduce opioid overdose deaths among people who use drugs: a systematic review and meta-analysis

**DOI:** 10.1186/s12889-025-22210-8

**Published:** 2025-03-25

**Authors:** Leah S. Fischer, Alice Asher, Renee Stein, Jeffrey Becasen, Amanda Doreson, Jonathan Mermin, Martin I. Meltzer, Brian R. Edlin

**Affiliations:** 1 Present Address: National Center for Emerging and Zoonotic Infectious Diseases, Atlanta, GA United States of America; 2 National Center for HIV/AIDS, STD and TB Prevention, Viral Hepatitis, Atlanta, GA United States of America; 3 Present Address: National Center for Injury Prevention and Control, Atlanta, GA United States of America; 4 Present Address: Immediate Office of the Director, Atlanta, GA United States of America; 5 Present Address: Substance Abuse and Mental Health Services Administration, Rockville, MD United States of America

**Keywords:** Systematic review, Naloxone, Opioid, Injection drug use, Overdose, Prevention, Syringe services programs, Police

## Abstract

**Background:**

It is estimated that over 111,000 people in the U.S. died from a drug overdose in the twelve-month period ending in July 2023. More than three-quarters of those deaths were attributed to opioids. Naloxone has long been available in healthcare facilities to reverse opioid overdose rapidly and safely but is not universally accessible for use in community settings where overdoses occur. We conducted a systematic literature review and meta-analysis to assess the effectiveness of overdose education and naloxone distribution (OEND) programs in three types of community settings to reduce overdose deaths among people who use opioids nonmedically.

**Methods:**

We systematically searched electronic databases, including Medline (OVID), Embase (OVID), Psycinfo (OVID), and Global Health (OVID), for peer-reviewed studies of OEND programs published during 2003–2018 (Group 1) that reported overdose outcomes individual level survivals or deaths immediately following naloxone administration. The PRISMA checklist guided screening, quality assessment, and data abstraction. We later identified studies published during 2018–2022 (Group 2), when drug usage and fentanyl-related overdose deaths notably increased, differed from earlier ones. We conducted meta-analyses on both Groups using random effects models to estimate summary survival proportions.

**Results:**

Among the 44 Group 1 studies published during 2003–2018, survival did not differ by time (year), location, naloxone dose, or route of administration, but studies of OEND programs serving people who use drugs reported 98.3% (95% CI: 97.5–98.8) survival; those serving family of people who use drugs or other community members reported 95.0% (95% CI: 91.4–97.1) survival; and those for police reported 92.4% (95% CI: 88.9–94.8) survival (*p* < 0.01). Five Group 2 studies (2018–2022) yielded similar results.

**Conclusions:**

Community-based naloxone distribution programs can be effective in preventing opioid overdose deaths. The paper demonstrates that in the face of increasing overdose deaths over time, survival after naloxone administration has been sustained. The very high survival rates provide clear evidence for public health to continue efforts to expand channels for naloxone distribution in community settings.

**Supplementary Information:**

The online version contains supplementary material available at 10.1186/s12889-025-22210-8.

## Introduction

In the United States, drug overdose is one of the leading causes of premature death, and the number of opioid-involved overdose deaths has increased steadily since 1990 [1, 2, 3]. From 2015 to 2021, the number of opioid deaths increased almost 143%, from 33,091 to 80,411 per year [2]. 

Death from opioid overdose is preventable through the timely provision of naloxone [4], a safe, non-addictive opioid antagonist that can rapidly reverse opioid overdose, restoring spontaneous respiration and consciousness. Naloxone has long been available in healthcare facilities to reverse opioid overdose rapidly and safely but is not universally accessible for use in community settings where overdoses occur [5.^6^].

Nonfatal opioid overdoses are often witnessed by companions who also use opioids [7]. In the 1990s, community activists developed programs distributing naloxone to people who use drugs, often as a part of syringe services programs (SSPs), as a response to the rapid rise in overdose deaths [8, 9]. Stigma, lack of resources, and legal barriers, however, limited implementation of naloxone distribution for many years [6, 10, 11]. 

Over the years, as evidence accumulated regarding the safety and effectiveness of naloxone administration, overdose education and naloxone distribution (OEND) programs have grown in number [12], and state and local public health agencies in some locations have joined or supported community-based programs distributing naloxone to people who use drugs, their peers, and partners [13, 14]. A 2019 online survey of U.S.-based SSPs showed a ten-fold increase in the number of OEND programs over the previous decade [12]. The majority of naloxone distribution, however, was delivered by only 14 SSPs, underscoring how severely limited existing OEND programs are in number, scope, and coverage. A recent study of naloxone needs in the United States found that the extent of naloxone distribution warrants substantial expansion in nearly every US state [15]. 

To inform decisions in public health planning about how best to deploy resources to naloxone distribution to maximize overdose survival. We, therefore, conducted a systematic review and meta-analysis to assess the existing evidence on the effectiveness of distributing naloxone to people who use drugs and other lay groups to reduce overdose deaths among people who use opioids nonmedically. We defined effectiveness as survival immediately after naloxone administration. This review updates previous reviews [16, 17, 18, 19, 20, 21], which generally found naloxone to be successful at reversing overdoses, and expands the literature base by adding a meta-analysis comparing the effectiveness of naloxone distribution across three distinct populations: people who use drugs (PWUD), family and friends of PWUD and other community members, and police. Additionally, because of recent upsurges in drug use and fentanyl-related opioid overdose deaths [2, 3, 22],we compared findings in recent years (2018–2022) with those from the previous 15 years (2003–2018).

## Materials and methods

This review encompasses data from two time periods. We conducted the initial literature search across multiple databases encompassing papers published from 2003 to 2018 (Group 1). To examine the previously mentioned potential impact of post-2018 upsurges in drug use and fentanyl-related opioid overdose deaths, we updated the search for papers published between 2018 and 2022 (Group 2). For both searches, we captured publication dates of the included studies as study years were often long and overlapping. We used publication date because this indicates when reported data were available for policy-making. No protocol was registered for the review.

### Group 1 data sources, 2003–2018

We conducted an electronic literature search for articles published from January 2003 through June 2018 in the following databases (platforms): Medline (OVID); Embase (OVID); Psycinfo (OVID); Global Health (OVID); CINAHL (EBSCO); NTIS (EBSCO); Scopus; and Cochrane Library (See Additional file 1, Supplement, Table S1). We also manually searched the reference lists of four published literature reviews [16, 17, 18, 19]. 

### Group 2 data sources, 2018–2022

Because most of the overdoses reported in Group 1 studies occurred before fentanyl-related overdose deaths began increasing rapidly in 2015 [22], we repeated the search for articles published from July 2018 through December 2022 (Group 2) in the PubMed system using the same search terms and inclusion and exclusion criteria and compared findings from the two time periods. We used only PubMed for this update because > 90% of the 44 papers in the 2003–2018 group (See Additional file 1, Supplement, Table S2) are indexed in PubMed.

### Study inclusion and exclusion criteria for groups 1 and 2

We limited the review to peer-reviewed reports of studies published in English from January 2003 through December 2022, conducted in the United States, Canada, Australia, or Europe, that reported on community-based programs, such as harm reduction programs, that provided OEND to lay persons (people without professional or specialized knowledge in substance use disorder or healthcare). We included only studies that provided descriptions of the study population, the intervention(s), and our primary outcome measures. We excluded studies of interventions based in healthcare settings such as the emergency department and pharmacies, solely qualitative studies, reviews, case studies/series, dissertations, letters, and editorials. There were no screening criteria related sample size or study design. A national survey reporting overdose outcomes from OEND programs across the United States [13] was excluded because some of the data duplicate those reported by some studies in this review.

### Outcomes for groups 1 and 2

The primary outcome of interest was reported individual level survivals or deaths immediately following naloxone administration. We considered reports of overdose “reversals,” “successful reversals,” “rescues,” and “survivals” as survivals, and reports of “deaths” or “unsuccessful” interventions as deaths. Additional outcomes abstracted as available were number of persons who received overdose training and naloxone, number of naloxone kits distributed, number of study participants, the number of persons with follow-up data (if they returned, for example, for a refill, prospectively scheduled follow-up, or another reason), and the number of overdoses in which participants intervened to administer naloxone. Follow-up was considered passive when naloxone use was ascertained only if participants returned to the program to obtain a naloxone refill or for another reason. When studies did not specify the number of participants with follow-up data, we used other information authors reported as minimum estimates to infer this value, in the following order: 1) the number of participants who reported using naloxone; 2) the number of overdoses for which participants reported using naloxone; or 3) number of naloxone reversals reported. We also identified a small sub-group (N = 5) of papers that reported community-levels of incidence of naloxone-related survival following life-threatening overdoses. The data in these papers could not be used in our statistical meta-analyses, but we qualitatively examined and reported their outcomes.

### Group 1 study selection and data extraction, 2003–2018

We used web-based systematic review software (DistillerSR, Evidence Partners, Ottawa, Canada) to create forms, screen articles, and extract data. The lead reviewer and one or two additional reviewers screened all titles and abstracts using a structured form. One reviewer was needed to include a study and two were required to exclude a study. Two reviewers then independently reviewed all full-text articles using a structured data abstraction form to record intervention characteristics, study characteristics, and outcomes. Reviewers reconciled discordant screening assessments and data abstraction conflicts though joint review and discussion, consulting a third team member when necessary for resolution.

### Group 2 study selection and data extraction, 2018–2022

Using PubMed for the Group 2 literature search, we applied the same selection criteria that we used for the Group 1 search. As mentioned above, we used only PubMed for the update because > 90% of the 44 papers in the 2003–2018 group (See Additional file 1, Supplement, Table S2) are indexed in PubMed. Rather than using Distiller software to abstract data, for the update, we manually abstracted data directly into an Excel spreadsheet.

### Group 1 study quality assessment, 2003–2018

The Community Guide methodology to assess intervention effectiveness informed our approach to assess study quality [23]. We tailored a set of ten Likert-scale questions in the following five domains to meet the needs of the review for studies reporting individual-level outcomes: description of the program being evaluated and the study methods, sampling, measurement of outcomes, data analysis, and interpretation of results. We report results separately for each question because assigning a value to the relative importance of each item would have been arbitrary or subjective [24]. No study was excluded from the analysis because of poor study quality.

### Group 2 study quality assessment, 2018–2022

We did not assess the quality of the Group 2 studies due to the paucity of identified studies. Similar to the methodology used for Group 1, no Group 2 paper was excluded due to poor quality.

### Data analyses for groups 1 and 2

Data extracted from studies reporting individual-level data were exported to Excel for data synthesis and analysis; we separately summarized and reported qualitatively the small sub-set of studies reporting community-level data. We calculated the proportion of reported survivals per naloxone use, defined as the proportion of people who remained alive after receiving naloxone among all people for whom an outcome was reported. In other words, the outcome metric was the calculated proportion of people surviving after receiving naloxone, which was the number of reported “survivals” as the numerator and the total number of reported outcomes as the denominator. Survival proportions are reported separately and compared for programs that enrolled PWUD, family or friends of PWUD and other community members, and police. When studies reported separately on multiple groups, such as PWUD and family members, we examined the data on each group separately.

### Meta-analyses for groups 1 and 2

Meta-analyses were conducted separately for each Group, using survival as the outcome variable. Group survival proportions and 95% confidence intervals (CIs) were calculated for all studies overall and were compared among subgroups by the following variables: Population who received OEND (PWUD, family of PWUD or community members, police), location (US vs. non-US), time (year), naloxone dose and route of administration (injected vs. nasal), and training duration. Comparisons between groups were conducted using the Comprehensive Meta-Analysis software (Biostat, Englewood, NJ). For subgroup comparisons, we used random effects models to calculate summary estimates of survival, the DerSimonian–Laird methods to estimate between-study variance, and the inverse-variance to weight each study. An alpha of 0.05 was used to assess statistical significance.

### Sensitivity analysis and robustness

We evaluated conducting a sensitivity analysis using only studies that had “high follow-up” and another sensitivity analysis that would exclude studies deemed to be of “poor quality.” We also evaluated robustness of our results by first focusing on studies with larger sample sizes (> 1,000 enrolled participants). Larger sample sizes generate smaller standard errors and smaller degrees of uncertainty (i.e., smaller confidence intervals). We also evaluated robustness by producing 2 “funnel plots” that analyzed the potential degree of publication bias and a “trim-and-fill” funnel plot to estimate the potential impact of “missing” studies. The first funnel plot charts the estimates of effectiveness (i.e., % survival) of each study against that study’s variance (precision). The trim-and-fill plot then examines the potential effect of including representative estimates of sizes and variance that the first funnel plot indicates may be missing from the included studies.

### Reporting

We used the Preferred Reporting Items for Systematic Reviews and Meta-Analyses (PRISMA) reporting checklist to guide the reporting of this review [25]. (See Additional file 2, PRISMA checklist).

## Results

### Group 1 article search, 2003–2018

**Fig. 1:**
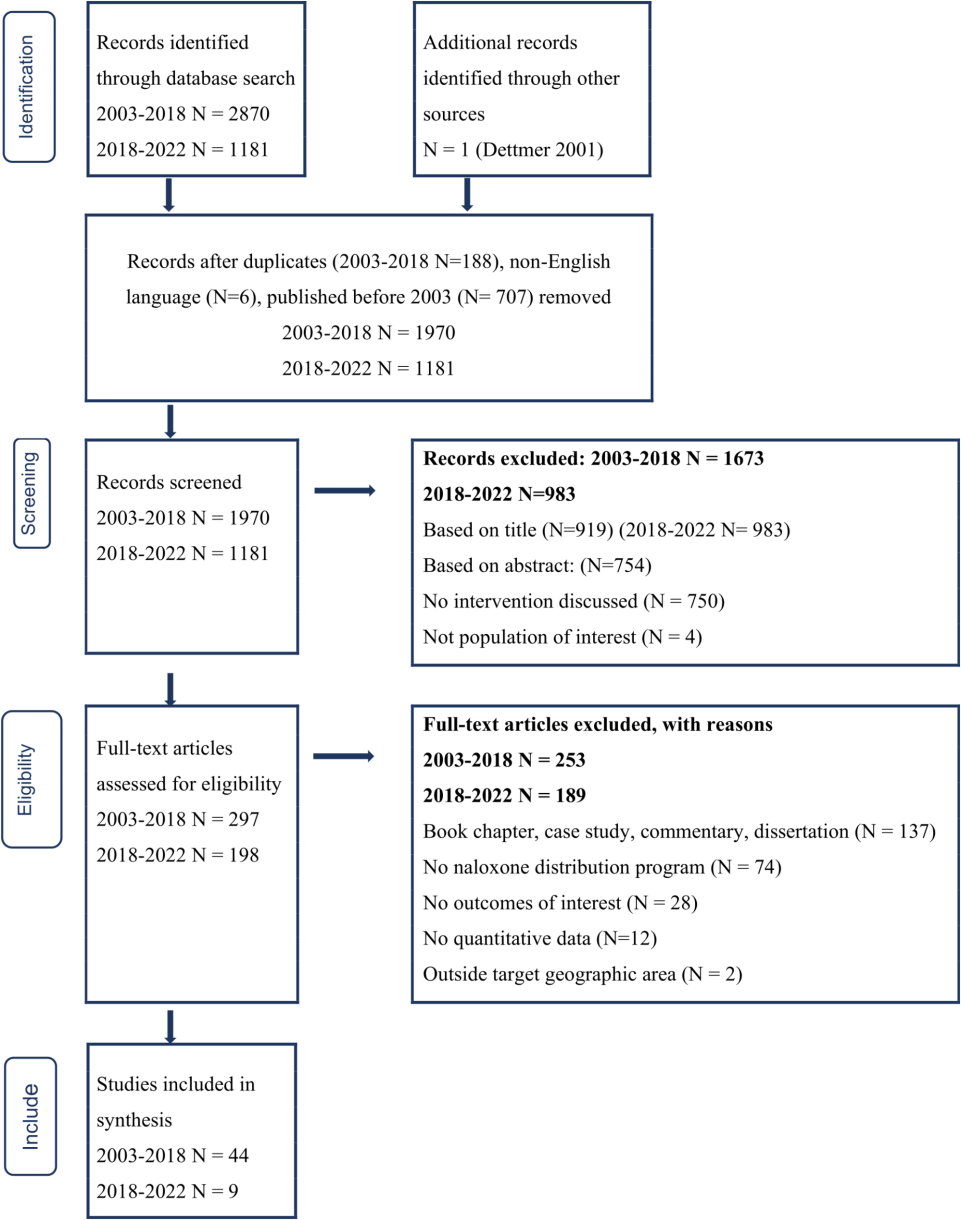
PRISMA flow diagram of study selection process for systematic review covering two time periods: 2003–2018 and 2018–2022

A CDC library-assisted search yielded 2,870 citations published from January 2003 through June 2018. After duplicates were removed, we identified 297 citations for full-text review and of these, determined 44 met inclusion criteria including one study identified from a manual search of existing literature review reference lists (See Fig. 1 and Additional file 1, Supplement, Table S2). Three review papers included only one study – Dettmer (2001) – published before 2003 and for this reason we included it. We report on 41 studies of OEND programs with individual-level outcome data and five studies with community-level data, including two that reported both individual- and community-level outcomes (See Table 1).

**Table 1:** Characteristics of 44 studies in systematic review of Naloxone distribution programs (2003–2018)^

**Studies with individual-level outcome data**		41	
Population served*			
People who use drugs	30		
Family, friends, others	6		
Law enforcement personnel	7		
**Location**			
United States	29		
Canada	5		
United Kingdom	5		
Other Europe†	2		
**Follow-up method**			
Passive	18		
Prospectively scheduled	12		
Only participants with follow-up included	4		
Not specified (law enforcement personnel studies)	7		
**Type of naloxone used**			
Injected	25		
Nasal	14		
Both	2		
**Studies with community-level outcome data**		**5**	
**Location**			
United States	4		
United Kingdom	1		
**Total studies‡**		**44**	

^See Table 3 for 2018–2022 data. *Two U.S. studies reported on programs for both people who use drugs and family members. †Other Europe = Norway, Germany, Channel Islands. ‡Two U.S. studies reported both individual- and community-level data

### Group 2 article search, 2018–2022

A PubMed search yielded 1,181 citations published in English from July 2018 through December 2022, from which 198 titles were selected for abstract screening. From a review of abstracts, we identified nine studies that met the original review criteria, excluding 189 because they were not community-based studies of naloxone distribution programs, did not report outcome data, or were qualitative studies (See Fig. 1).

### Group 1 studies reporting individual-level outcomes (*N* = 41), 2003–2018

#### Group 1 study characteristics

The 41 studies with individual-level data were conducted in the U.S. (*n* = 29), Canada (*n* = 5), and Europe (*n* = 7) and delivered OEND to people who used drugs (*n* = 30), family, friends, and others (*n* = 6), and police (*n* = 7) (See Table 1). Median study size was 316 participants (range, 91–40,801; interquartile range, 99–1,322). Injectable naloxone was distributed in 25 studies, nasal naloxone in 14, and both in 2 (See Additional file 1, Supplement, Table S4). The median duration of OEND training was 30 min, ranging from 2 min to 8 h (See Additional file 1, Supplement, Table S4).

Intervention characteristics changed over time, reflecting the evolution of innovations in program practices and policies. (See Additional file 1, Supplement, Table S6). Early programs distributed injectable naloxone and sterile syringes at SSPs to people who injected drugs; later programs distributed nasal naloxone and began enrolling family members, other community members, and, still later, law enforcement personnel (See Additional file 1, Supplement, Tables S4 and S6). After 2007, program venues expanded beyond SSPs to include settings such as homeless shelters, community centers, and correctional facilities. OEND programs designed for police in our review were first implemented in 2013 [26]. Training duration declined over time, as programs switched to nasal naloxone (See Additional file 1, Supplement, Table S6).

#### Group 1 study quality, 2003–2018

Overall, most reports contained clear descriptions of the population served (*n* = 36); more than half provided a thorough or very thorough description of OEND training, including information about naloxone preparation (*n* = 23) (See Additional file 1, Supplement, Table S3). Most papers contained clear descriptions of study methods with respect to study design (*n* = 32), study participant sampling (*n* = 29), and specification of outcomes (*n* = 31). Six studies described how authors addressed potential selection bias arising from participant sampling. Reporting of outcomes was considered clear in 20 of the 41 studies and complete in 15; 10 did not specify certain denominators, such as total number of participants or numbers of kits distributed (See Additional file 1, Supplement, Table S3). Investigators reporting the findings in 14 studies were somewhat independent or strictly independent from program staff carrying out the intervention, and not very or not at all independent in 17 studies. The relationship was not described, or we could not tell in 10. Eight of 41 studies did not discuss study limitations. Overall, most of the studies were categorized as being “good quality.”

#### Group 1 data reporting follow-up, 2003–2018 (41 studies)

Participant follow-up varied by study and follow-up methods used. In many studies follow-up was passive; follow-up data were collected only when participants returned for refills or to receive other services. When follow-up was prospectively scheduled, the interval ranged from two to 12 months, with a three-month interval utilized most frequently [27–32] The median proportion of participants with follow-up data was 10.5% among 18 studies that used passive follow-up but 74.5% in 12 studies with prospectively scheduled follow-up. Four studies, by design, included only participants with follow-up data, and follow-up was not described in the seven police studies.

### Group 1 individual-level outcomes, 2003–2018

The 41 studies reported individual-level data on 74,114 study participants, who reported outcomes (survival or death) after 10,328 naloxone administrations (See Table 2). Survival did not differ by location (US vs. other countries), time (year), training duration, or naloxone dose or route of administration. Survival estimates did differ, however, by the population to whom OEND was provided (See Table 2).

**Table 2:** Outcomes in 41 studies reporting individual-level data (2003–2018)^

	No. studies	No. Participants		Kits Distributed		Survivals Reported		Deaths Reported		Summary Survival Proportion (95% CI)*
**Population served**†										
People who used drugs	30	64,947		75,375		8,686		100		98.3% (97.5, 98.8)
Family, friends, others	6	7,868		8,414		239		10		95.0% (91.4, 97.1)
Police	7	1,299‡		1,556‡		1,200		93		92.4% (88.9, 94.8)
**Route of administration** ^§^										
Injection	25	15,990		19,506		3,106		15		97.9% (96.2, 98.8)
Nasal	14	56,322‡		63,288‡		6,292		178		97.7% (94.1, 97.9)
**Naloxone dose** (by injection)^¶^										
0.4 mg	16	13,467		16,056		2,653		13		98.2% (95.8, 99.2)
0.8 mg – 2.0 mg**	5	1,658		2,110		175		0		97.0% (94.1, 98.5)
**Total**	41†	74,114		85,345		10,125		203		97.3% (96.1, 98.2)

^See Table 3 for 2018–2022 data. *Proportion surviving among events with known outcome (survival or death) calculated using random effects models. †Two studies reported on data from both people who used drugs and other community members (family, friends, others). ‡Data missing (no. of participants and kits) for 3 studies. ^§^Two studies that used both nasal and injected naloxone are excluded. ^¶^Includes only studies that used injected naloxone and specified the dose. **The dose was 1.0 mg in 3 of the 5 studies

### Group 2 studies reporting individual-level outcomes, 2018–2022

Of the nine studies that met inclusion criteria, five were conducted in the US (four studies with individual-level data, one with county-level data), two in Scandinavia, one in Canada, and one in Australia (See Table 3). Among the Group 2 studies, survival proportions across groups that received OEND/administered naloxone were similar to that in the Group 1 studies (See Table 3). Survival in 4 studies of programs serving PWUD was 96-100%; in 3 studies of programs distributing naloxone to PWUD and other lay persons survival was 96-97%; and the single evaluation of a training program for police reported a 95% survival (See Table 3). In the only study in our review to enroll PWUD, family members, other community members, and police all in the same study, the odds of PWUD administering naloxone to someone to reverse an overdose were more than 8 times the odds that people in the other groups did so [33]. A ninth study examined changes in county-level overdose death rates before and after OEND implementation in the 38 North Carolina counties with the most opioid overdoses [34].

**Table 3:** Characteristics and outcomes for studies published during 2018–2022 (*N* = 9)

Author (Year)**	Location	Population served	No. participants	Survivals*		Deaths*		Included in meta-analyses
**Studies reporting individual-level data**								
Lintzeris (2020)	Australia	PWUD†	145	9	(100.0%)	0	(0.0%)	Yes
Buresh (2020)	Baltimore	PWUD	346	68	(95.8%)	3	(4.2%)	Yes
Jones (2022)	New York	PWUD	321	161	(97.0%)	5	(3.0%)	Yes
Troberg (2022)	Sweden	PWUD	165‡	123	(100.0%)	0	(0.0%)	Yes
Janssen (2020)	Michigan	Police	508	174	(94.6%)	10	(5.4%)	Yes
Williams (2021)	Canada	Community service providers	NR§	1,517	(98.5%)	23	(1.5%)	No
Yang (2021)	United States	Mixed¶	3,609	335	(97.4%)	9	(2.6%)	No
Thylstrup (2019)	Denmark	Mixed#	552	23	(95.8%)	1	(4.2%)	No
**Total**			5,646	2,410		51		
**Study reporting community-level data**								
Naumann (2019)	No. Carolina		352 overdose deaths averted/3 years					No

*Percentages are of events with known outcomes (survival or death). ** For full references, see Table S2. Articles included in systematic literature review on community-based naloxone distribution programs (2003–2022). †PWUD = People who use drugs. ‡Number of participants recruited from community-based locations. §NR = Not Reported. ¶Mixed = People who used drugs and other community members. #Mixed = People who used drugs, other community members, and law enforcement

#### Group 2 study quality and patient follow-up, 2018–2022

As detailed in Methods, there were so few studies that met inclusion criteria, we included all regardless of study quality. As in Group 1 (2003–2018) most of the studies were considered to be of “good quality.” However, of the nine studies that met the original review criteria, only 5 reported data sufficient to include in the meta-analysis. None of the studies provide sufficient details on patient follow-up after the initial use of naloxone.

#### Meta-analyses: survival for groups 1 and 2

**Fig. 2:**
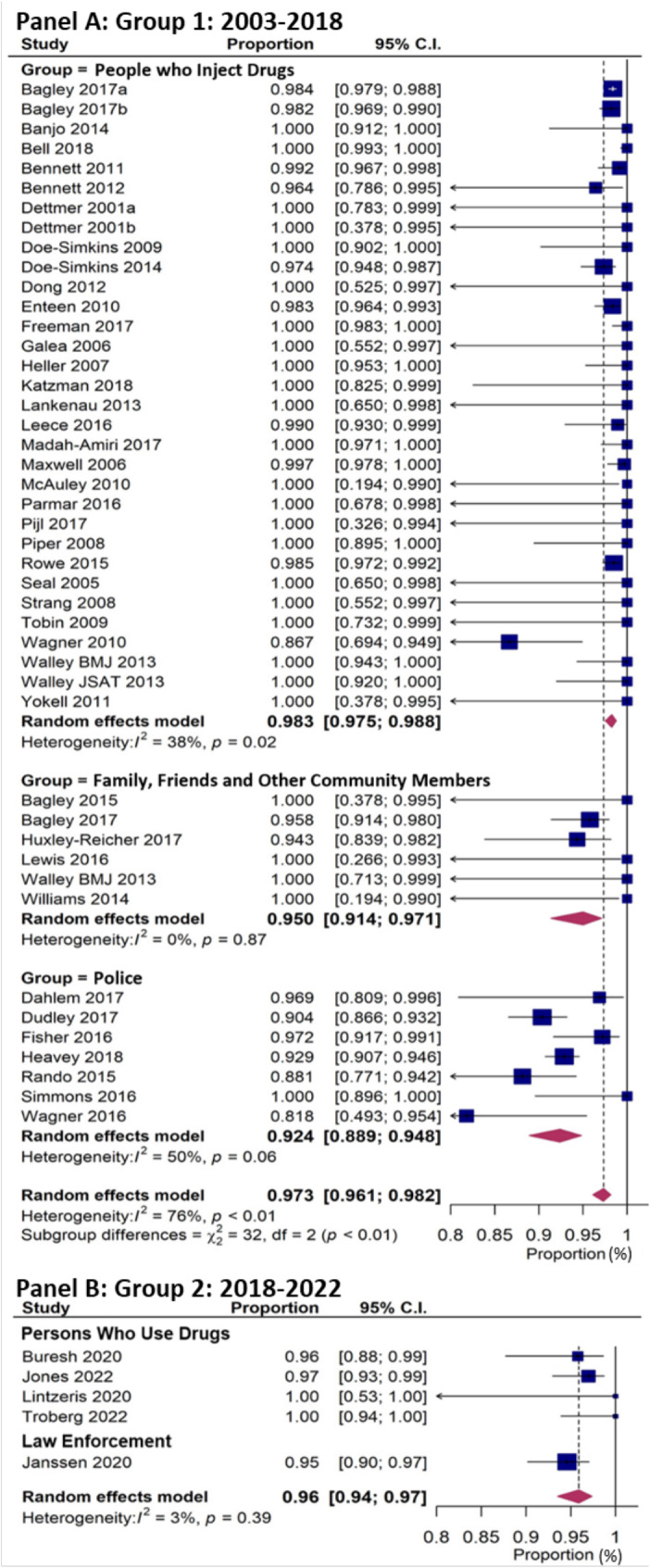
Forest plot of studies of naloxone distribution programs with individual-level survival data: **Group 1** (2003-2018) and **Group 2** (2018-2022). *Note: ‘Group’ refers to ‘study sample distributed naloxone’

Overall, from the random effects model for all Group 1 studies, 97.3% (95% CI: 96.1–98.3) of persons who received naloxone survived (SeFig. . 2). Average survival in Group 1 studies of programs serving people who use drugs was 98.3% (95% CI: 97.5–98.8); in programs serving family of people who use drugs or other community members, 95.0% (95% CI: 91.4–97.1); and in programs distributing naloxone to police, 92.4% (95% CI: 88.9–94.8) (SeFig. . 2). Considerable heterogeneity existed between all studies (I^2^ = 76%, *p* < 0.01), but decreased for studies categorized by group to whom naloxone was distributed: PWUD (I^2^ = 38%, *p* = 0.02); family/friends (I^2^ = 0%, *p* = 0.87); and law enforcement (I^2^ = 50%, *p* = 0.06) (See Fig. 2).

Similar to the results for Group1, studies in Group 2, 96.0% ((95% CI:94-97%) of persons who received naloxone survived (See Fig. 2). The meta-analysis of these 5 studies had a Heterogeneity I [2] of 3% (*p* = 0.39), indicating very little variance between studies. The *p* value of 0.39 is likely due to the wide confidence intervals measured in the Lintzeris et al. study (See Table 3; Fig. 2). These results from Group 2 indicate that the post-2018 upsurges in drug use and fentanyl-related opioid overdose deaths did not negatively impact the effectiveness, at an individual level, of community and police-based naloxone programs.

#### Sensitivity analysis and robustness

We found, for two reasons, that it was technically not feasible to conduct a sensitivity analysis using only studies that had “high follow-up” of OEND program participants that returned, for instance, for a refill of naloxone. The first reason is that there were only 10 studies, with varying timelines, out of the original 44 studies (2003–2018) that reported active follow-up protocols. And those that reported had differing periods of follow-up ranging from 2 months to 8 months (See Additional file 1, Supplement, Table S5). The remainder reported “passive” follow up. The second problem was some of those that had an active follow-up period had very small enrollments. For example, Seal (2005) had 25 participants enrolled (with a 6-month follow-up), Galea (2006) had 25 participants (3-month follow-up) and Wagner (2010) had 66 participants enrolled (3 month follow up) (See Additional file 1, Supplement, Table S5). For sensitivity analysis that would exclude studies deemed to be of “poor quality,” most of the studies were evaluated as being of “good quality” (described earlier). A sensitivity analysis that focused on only those of good quality would basically repeat the analysis already done (See Fig. . 2).

For evaluating robustness, considering only the 10 studies that enrolled at least 1,000 participants (See Additional file 1, Supplement, Table S5), it can readily be seen in Fig. 2 that those studies have higher averages, and smaller confidence intervals, than the overall average (i.e., the plots for those 10 studies are “to the right” of the red diamond, which plots the average for all studies). We therefore conclude that our results are robust. Using funnel plots to further examine robustness, we found that our first funnel plot (See Additional file 1, Supplement, Figure S1A) indicated that there may be missing studies with lower survival and higher variance (bottom left-hand quadrant) [35]. The trim-and-fill funnel plot added in 6 imputed “missing” studies (plotted as white circles in Additional file 1, Supplement, Figure S1B). Adjusting overall summary estimates, no substantial impact of publication bias on our summary estimates was observed. The original estimate of survival was 97.3% (95% CI: 96.1–98.3); with adjustment using trim-and-fit methodology, it was 96.5% (95.0–97.6).

### Studies reporting community-level outcomes (*N* = 5)

Five studies reported community-level overdose death data where naloxone distribution programs were instituted. Two of these also reported individual-level data and were included in the analysis presented above.

A report on an OEND program that distributed > 3,500 naloxone vials to people who injected drugs in Chicago included Cook County overdose data showing a 2.4-fold rise in annual overdose deaths during the 4 years before the program started in January 2001, followed by a 30% reduction in deaths during the 3 years after the program began [36].

An interrupted time series analysis examined opioid overdose mortality rates in communities in Massachusetts with no OEND implementation, low OEND implementation (1–100 people trained per 100,000 population) and high OEND implementation (> 100 people trained per 100,000 population). After adjusting for community level demographics and substance use factors, authors found 27% and 46% reductions in opioid overdose mortality rates in the low- and high-implementing communities, respectively, when compared with communities with no OEND implementation [37].

A spatial analysis examined the locations of 316 opioid overdose reversals and 342 overdose deaths in San Francisco. Naloxone distribution sites were located in areas with greater numbers of drug arrests and historically endemic levels of substance use. In analyses that controlled for other factors, geographic proximity to a naloxone distribution site was associated with more frequent overdose reversals by means of naloxone administration, but not opioid overdose deaths [38].

In January 2011, Scotland began providing naloxone to prisoners on release from its prisons. A before (2006-10) -after (2011-13) comparison of Scotland’s National Naloxone Programme found a 36% reduction in the proportion of opioid-related deaths in prisoners during the 4 weeks following their release from prison after the program was initiated [39]. These results led to the discontinuation of a randomized trial underway in English prisons of naloxone on release, and the provision of naloxone to all participants [40].

Investigators in San Francisco reported on the response of the city’s naloxone distribution program to the introduction of fentanyl into the local illicit drug supply, which had been associated with large increases in drug overdoses in communities across the nation [41]. In San Francisco, the program intensified community outreach and naloxone distribution as soon as fentanyl was detected. New participant enrollment increased 55%, naloxone refills increased 89%, and reported overdose reversals increased 113% compared with the same timeframe the previous year (all *p* < 0.001), but there was no significant increase in the number of opioid-involved overdose deaths. The authors concluded that the naloxone distribution program may have helped avert the large increases in drug overdose deaths that accompanied the introduction of illegally made fentanyl elsewhere in the United States.

## Discussion

Naloxone is a life-saving medication capable of reversing potentially fatal opioid overdoses. Our systematic review revealed high rates of successful resuscitation by lay persons in 41 studies, including people who used drugs, their friends and family and other community members, and police. In all, more than 10,000 successful overdose reversals were reported. Our review found high survival rates (93-98%) following the use of naloxone when persons trained and administering the medication were PWUD, family members, or police. A national survey of naloxone distribution programs in the United States, not included in this review because of potentially overlapping data, told the same story: naloxone distributed in 136 programs resulted in more than 26,000 reported overdose reversals [13]. 

These studies included no comparison groups, so how many of these overdoses would have resulted in death had OEND not been implemented is unknown. However, six studies in our review using pre-post designs, an interrupted time series analysis, and a spatial distribution analysis assessed the effects of OEND programs on local overdose rates in Chicago, Massachusetts, San Francisco, Scotland, and North Carolina. All six studies found evidence of the programs’ effectiveness, including substantial reductions in overdose mortality (25-46%) in three of the studies. The sixth study estimated that naloxone distribution in North Carolina averted 352 overdose deaths over a three-year period, at a cost of $1,605 per life saved [34].

While 1.7% of overdosing persons died in studies when naloxone was distributed to PWUD, 5.0% died when it was distributed to family, friends, and other community members, and 7.6% died when it was distributed to police. Possible reasons for differences could be proximity to the overdose event and time to naloxone administration. PWUD are more likely than family members or police to be present or nearby at the time of overdose and able to administer naloxone in the early stages of overdose onset. They are often experienced in the use of needles, syringes, and percutaneous injection, and most have prior experience witnessing overdose [7]. While survival after law enforcement naloxone administration was high (92.4%), it was the lowest of the three groups, likely because emergency services are often not summoned until an overdose has progressed to late stages [42, 44]. Evidence also indicates that police may feel less confident, after training, in administering naloxone than other groups [45].

Comprehensive community-based programs that include syringe services programs integrated with linkage to medication to treat opioid use disorder, naloxone distribution, HIV care, and viral hepatitis testing and treatment are likely to be particularly effective in the mutual goals of preventing overdose deaths, reducing substance use, and preventing infectious diseases. A modelling study that examined the cost-effectiveness of naloxone distribution through SSPs for people who use drugs with or without treatment for substance use disorder found naloxone distribution alone was cost-effective at $323 per quality-adjusted life year, and with the addition of linkage to treatment for substance use disorders was cost-saving [46].

Our review is subject to several limitations. First, follow-up was low in many studies. Our analysis most likely underestimates overdose event outcomes. Studies often included only reversals reported by people who returned for refill or were in a prospective study, excluding reversals that may have been performed by participants who did not return and report their experiences. Both successful and unsuccessful naloxone administrations were likely underreported, and we did not examine possible outcomes that occur after initial survival. Second, our findings could be subject to publication or small study bias. People implementing programs may be more motivated to report successes than failures. Our funnel plot and “trim-and-fill” analyses, however, yielded little evidence of such bias (Figure S1B). We did not review unpublished reports, or program or health department reports not published in scientific literature, whose findings might differ from the studies we reviewed. Third, many of the evaluations were not independent; often they were conducted by people involved in running the programs or collaborating with them. Fourth, overdoses reversed do not necessarily translate into reduced overdose deaths, because some people may have survived without receiving naloxone or after receiving it from emergency medical services. Findings from six studies reporting community-level overdose rates, however, consistently reported lifesaving effects of community-based naloxone distribution programs. Finally, after a search timeframe for a review ends, potentially relevant papers are published on an ongoing basis. For example, using the original search strategy, a search in PubMed found three papers published during Jan. 2022-Oct. 2024 that met the review criteria [47–49]. These studies contained no notable differences in conclusions than those found in this review.

As opioid overdoses increasingly involve synthetic opioids such as illegally made fentanyl including fentanyl analogs and the combined usage of opioids together with benzodiazepines or other sedatives, it becomes increasingly important to be sure naloxone is available when and where it is needed so it can be used as soon as possible. Further, as fentanyl contamination is increasingly found in drugs sold as methamphetamine, cocaine or other drugs not identified as an opioid [50], it is important to ensure that all PWUD know how to identify an overdose and administer naloxone to save the lives of their peers. Ensuring PWUD, particularly populations disproportionately impacted by overdose, have access to this life-saving intervention is critical as overdose deaths remain at historic levels [2]. Including PWUD as equal partners in the design and execution of overdose prevention efforts is necessary for these efforts to be successful [8, 14]. 

Our findings support previously published studies regarding the effectiveness of widespread naloxone distribution to people who use illicit drugs [10, 15,51–54]. Distribution of naloxone to people who use drugs has been constrained by several barriers, including stigma, [10, 55, 56], insufficient funding [15,57], legal constraints [58, 59], naloxone pricing [60, 61]. supply chain shortages [62], manufacturing problems [63, 64], and regulatory processes [65].

Efforts to overcome these barriers are underway. Community members, researchers, service providers, and non-profit organizations have created novel pathways, along with federal partners, to provide naloxone at low cost to under resourced community programs providing OEND to people who use drugs [57, 66–68]. The Food and Drug Administration has taken steps to relieve regulatory impediments encumbering naloxone distribution through nonmedical community organizations [69] and has approved an over-the-counter nasal naloxone device [70] made by a non-profit manufacturer who will distribute it to programs in need at low or no cost [71, 72]. The White House has put forth model naloxone access laws for states to consider [73] and has met with naloxone manufacturers to urge them to increase naloxone access and affordability for at-risk communities and organizations serving high-risk individuals [74]. The Substance Abuse and Mental Health Administration funds states, tribes, and communities to purchase naloxone and develop plans to saturate their communities with naloxone. The Centers for Disease Control and Prevention funds OEND through the Overdose Data to Action in States and Localities programs [75] and provides implementation toolkits and other materials through the Stop Overdose campaign [14, 76], Concerted action by community members, nonprofit organizations, governments, and industry, partnering with people who use drugs, can make substantial progress to close the gap toward making naloxone available when and where it is needed to reduce overdose deaths.

## Conclusions

Community-based naloxone distribution programs can be effective in preventing opioid overdose deaths. The paper demonstrates that in the face of increasing overdose deaths over time, survival after naloxone administration has been sustained. The very high survival rates provide clear evidence for public health to continue efforts to expand channels for naloxone distribution in community settings.

## Electronic supplementary material

Below is the link to the electronic supplementary material.

Supplementary Material 1

Supplementary Material 2

## Data Availability

All data generated or analysed during this study are included in this published article and its supplementary information files.

## Abbreviations

OEND: Overdose education and naloxone distribution
PWUD: People who use drugs
CIs: Confidence intervals
