# Supplementary material for: Effectiveness of naloxone distribution in community settings to reduce opioid overdose deaths among people who use drugs: a systematic review and meta-analysis
Source: BMC Public Health. 2025 Mar 25;25:1135. doi: 10.1186/s12889-025-22210-8 (PMC11934755; doi:10.1186/s12889-025-22210-8)
Supplement: Supplementary file 1 — Supplementary Material 1 [file 12889_2025_22210_MOESM1_ESM.docx]

**Additional file 1. Supplement: Systematic literature review and meta-analysis of effectiveness naloxone distribution (OEND) in community settings in reducing opioid overdose deaths**

**CONTENTS**

1. [Table S1. Search strategy](#OLE_LINK1)
2. [Table S2. Articles included in systematic literature review (2003-2022)](#OLE_LINK2)

1. [Table S3. Summary of Quality of Execution (QoE) items in 41 studies with individual-level data, 2003-2018](#OLE_LINK3)
2. [Table S4. Intervention characteristics of community-based naloxone distribution programs in 41 studies with individual-level outcome data, 2003-2018](#OLE_LINK4)
3. [Table S5. Study characteristics and selected outcomes of community-based naloxone distribution programs](#OLE_LINK5)

[Additional findings](#OLE_LINK6)

1. [Table S6. Characteristics of studies of naloxone distribution programs: Secular trends over time (N=41)](#OLE_LINK6)
2. [Figure S1A:](#OLE_LINK7) [Funnel plot to check for the existence of publication bias](#OLE_LINK7)
3. [Figure S1B: Funnel plot with trim-and-fill to estimate potential impact of possible missing studies](#OLE_LINK7)

**[Table S1. Search strategy](#OLE_LINK1)**

**Search Query:** Take-home naloxone distribution programs to reduce opioid overdose deaths among people who use drugs (PWUD)

**Search Strategy:**

| **Database** | **Strategy** | **Run Date** | **Records** |
| --- | --- | --- | --- |
| **Medline**  **(OVID)**  **1946-** | Naloxone* OR Narcan OR Narcanti OR nalone OR mrz 2593* OR mrz2593*  AND  Overdose* | 6/5/2018 | 1041  -85 duplicates  =946 unique items |
| **Embase**  **(OVID)**  **1947-** | Naloxone* OR Narcan OR Narcanti OR nalone OR mrz 2593* OR mrz2593*  AND  Overdose* | 6/5/2018 | 2091  -589 duplicates  =1502 unique items |
| **PsycInfo**  **(OVID)**  **1967-** | Naloxone* OR Narcan OR Narcanti OR nalone OR mrz 2593* OR mrz2593*  AND  Overdose* | 6/5/2018 | 330  -233 duplicates  =97 unique items |
| **Global Health**  **(OVID)**  **1973-** | Naloxone* OR Narcan OR Narcanti OR nalone OR mrz 2593* OR mrz2593*  AND  Overdose* | 6/5/2018 | 102  -59 duplicates  =43 unique items |
| **CINAHL**  **(Ebsco)** | Naloxone* OR Narcan OR Narcanti OR nalone OR mrz 2593* OR mrz2593*  AND  Overdose*  Exclude Medline Records | 6/5/2018 | 53  -15 duplicates  =38 unique items |
| **NTIS**  **(Ebsco)** | Naloxone* OR Narcan OR Narcanti OR nalone OR mrz 2593* OR mrz2593*  AND  Overdose* | 6/5/2018 | 4  -0 duplicates  =4 unique items |
| **Scopus** | Citation mapping – cited and cited by:  Clark, A.K., Wilder, C.M., Winstanley, E.L., 2014. A systematic review of community opioid overdose prevention and naloxone distribution programs. J. Addict. Med. 8, 153–163.  EMCDDA, 2015. Preventing Fatal Overdoses: A Systematic Review of the Effectiveness of Take-Home Naloxone. (Accessed 1 June2015). http://www.emcdda.europa.eu/  McDonald, R., Strang, J., 2016. Are take-home naloxone programmes effective? Systematic review utilizing application of the Bradford Hill criteria. Addiction 111, 1177–1187. | 6/5/2018 | 190  -20 duplicates  =170 unique items |
| **Cochrane Library** | (Naloxone* OR Narcan OR Narcanti OR nalone OR mrz 2593* OR mrz2593*):ti,ab  AND  (Overdose*):ti,ab | 6/5/2018 | 90  -20 duplicates  = 70 unique items |

Notes: Duplicates were identified using the Endnote automated "find duplicates" function with preference set to match on title, author and year, and removed from your Endnote library. There will likely be additional duplicates found that Endnote was unable to detect.

**[Table S2. Articles included in systematic literature review on community-based naloxone distribution programs (2003--2022)](#OLE_LINK2)**

| **(Group 1: 2003-2018, N=44)**   1. Bagley SM, Forman LS, Ruiz S, Cranston K, Walley AY. Expanding access to naloxone for family members: The massachusetts experience. Drug and Alcohol Review. 2018; 37(4):480-486. 2. Bagley SM, Peterson J, Cheng DM, Jose C, Quinn E, O'Connor PG, Walley AY. Overdose Education and Naloxone Rescue Kits for Family Members of Individuals Who Use Opioids: Characteristics, Motivations, and Naloxone Use. Substance Abuse. 2015;36(2):149-54. 3. Bell A, Bennett AS, Jones TS, Doe-Simkins M, Williams LD. Amount of naloxone used to reverse opioid overdoses outside of medical practice in a city with increasing illicitly manufactured fentanyl in illicit drug supply. Substance Abuse. 2018;1-12. 4. Bennett AS, Bell A, Tomedi L, Hulsey EG, Kral AH. Characteristics of an overdose prevention, response, and naloxone distribution program in Pittsburgh and Allegheny County, Pennsylvania. Journal of Urban Health. 2011;88(6):1020-30. 5. Bennett T, Holloway K. The impact of take-home naloxone distribution and training on opiate overdose knowledge and response: An evaluation of the THN Project in Wales. Drugs: Education, Prevention and Policy. 2012;19(4):320-8. 6. Bird SM, McAuley A, Perry S, Hunter C. Effectiveness of Scotland's National Naloxone Programme for reducing opioid-related deaths: a before (2006-10) versus after (2011-13) comparison. Addiction 2016;111(5):883-91. doi: [10.1111/add.13265](https://doi.org/10.1111/add.13265). PMID: 26642424; PMCID: PMC4982071. 7. Dettmer K, Saunders B, Strang J. Take home naloxone and the prevention of deaths from opiate overdose: Two pilot schemes. British Medical Journal. 2001;322(7291):895-6. 8. Doe-Simkins M, Quinn E, Xuan Z, Sorensen-Alawad A, Hackman H, Ozonoff A, Walley AY. Overdose rescues by trained and untrained participants and change in opioid use among substance-using participants in overdose education and naloxone distribution programs: a retrospective cohort study. BMC Public Health. 2014;14:297. 9. Doe-Simkins M, Walley AY, Epstein A, Moyer P. Saved by the nose: bystander-administered intranasal naloxone hydrochloride for opioid overdose. American Journal of Public Health. 2009;99(5):788-91. 10. Dong KA, Taylor M, Wild CT, Villa-Roel C, Rose M, Salvalaggio G, Rowe BH. Community-based naloxone: A Canadian pilot program. CJAM Canadian Journal of Addiction Medicine. 2012;3(2):4-9. 11. Enteen LB, McLean R, Wheeler E, Huriaux E, Kral AH, Bamberger JD. Overdose prevention and naloxone prescription for opioid users in San Francisco. Journal of Urban Health. 2010;87(6):931-41. 12. Freeman LK, Bourque S, Etches N, Goodison K, O'Gorman C, Rittenbach K, Sikora CA, Yarema M. Alberta's provincial take-home naloxone program: A multi-sectoral and multi-jurisdictional response to overdose. Canadian Journal of Public Health Revue Canadienne de Sante Publique. 2017;108(4):e398-e402. 13. Galea S,Worthington N, Piper TM, Nandi VV, Curtis M, Rosenthal DM. Provision of naloxone to injection drug users as an overdose prevention strategy: Early evidence from a pilot study in New York City. Addictive Behaviors. 2006;31(5):907-12. 14. Heller DI, Stancliff S. Providing naloxone to substance users for secondary administration to reduce overdose mortality in New York City. Public Health Reports. 2007;122(3):393-7. 15. Huxley-Reicher Z, Maldijian L, Winkelstein E, Siegler A, Paone D, Tuazon E, Nolan ML, Jordan, MacDonald R, Kunins HV. Witnessed overdoses and naloxone use among visitors to Rikers Island jails trained in overdose rescue. Addictive Behaviors. 2017;16:16. 16. Katzman JG, Takeda MY, Bhatt SR, Moya Balasch M, Greenberg N, Yonas H. An innovative model for naloxone use within an OTP Setting: A prospective cohort study. Journal of Addiction Medicine. 2018;12(2):113-8. 17. Lankenau SE, Wagner KD, Silva K, Kecojevic A, Iverson E, McNeely M, Kral AH. Injection drug users trained by overdose prevention programs: Responses to witnessed overdoses. Journal of Community Health. 2013;38(1):133-41. 18. Leece P, Gassanov M, Hopkins S, Marshall C, Millson P, Shahin R. Process evaluation of the Prevent Overdose in Toronto (POINT) program. Canadian Journal of Public Health Revue Canadienne de Sante Publique. 2016;107(3):e224-e30. 19. Lewis DA, Park JuNyeong, Vail L, Sine M, Welsh C, Sherman SG. Evaluation of the overdose education and naloxone distribution program of the Baltimore student harm reduction coalition. American Journal of Public Health. 2016;106(7):1243-6. 20. Madah-Amiri D, Clausen T, Lobmaier P. Rapid widespread distribution of intranasal naloxone for overdose prevention. Drug and Alcohol Dependence. 2017;173:17-23. 21. Maxwell S, Bigg D, Stanczykiewicz KD, Carlberg-Racich S. Prescribing naloxone to actively injecting heroin users: a program to reduce heroin overdose deaths. Journal of Addictive Diseases. 2006;25(3):89-96. 22. McAuley AL, George; Woods M, Louttit D. Responsible management and use of a personal take-home naloxone supply: A pilot project. Drugs: Education, Prevention & Policy. 2010;17(4):388-99. 23. Banjo OM, Tzemis D, Al-Qutub D, Amlani A, Kesselring S, Buxton JA. A quantitative and qualitative evaluation of the British Columbia Take Home Naloxone program. CMAJ open. 2014;2(3):E153-61. 24. Parmar M, Strang J, Choo L, Meade AM, Bird SM. Randomized controlled pilot trial of naloxone-on-release to prevent post-prison opioid overdose deaths. Addiction (abingdon, england) [Internet]. 2016. Available from: <http://cochranelibrary-wiley.com/o/cochrane/clcentral/articles/429/CN-01253429/frame.html>. 25. Pijl EM, Bourque S, Martens M, Cherniwchan A. Take-home naloxone kit distribution: A pilot project involving people who use drugs and who are newly released from a correctional facility. Canadian Journal of Criminology and Criminal Justice. 2017;59(4):559-71. 26. Piper TM, Stanciff S, Rudenstine S, Sherman S, Nandi V, Clear A, Galea S. Evaluation of a naloxone distribution and administration program in New York City. Substance Use & Misuse. 2008;43(7):858-70. 27. Rowe C, Santos GM, Vittinghoff E, Wheeler, E, Davidson P, Coffin PO. Predictors of participant engagement and naloxone utilization in a community-based naloxone distribution program. Addiction. 2015;110(8):1301-10. 28. Rowe C, Santos GM, Vittinghoff E, Wheeler E, Davidson P, Coffin PO. Neighborhood-Level and Spatial Characteristics Associated with Lay Naloxone Reversal Events and Opioid Overdose Deaths. Journal of Urban Health. 2016;93(1):117-30. 29. Rowe C, Wheeler E, Stephen Jones, T, Yeh, C, Coffin PO. Community-Based Response to Fentanyl Overdose Outbreak, San Francisco, 2015. Journal of Urban Health 2019;96(1):6-11. doi: 10.1007/s11524-018-0250-x. 30. Seal KH, Thawley R, Gee L, Bamberger J, Kral AH, Ciccarone D, Downing M, Edlin BR. Naloxone distribution and cardiopulmonary resuscitation training for injection drug users to prevent heroin overdose death: a pilot intervention study. Journal of Urban Health. 2005;82(2):303-11. 31. Strang J, Manning V, Mayet S, Best D, Titherington E, Santana L, Offor E, Semmler C. Overdose training and take-home naloxone for opiate users: prospective cohort study of impact on knowledge and attitudes and subsequent management of overdoses. Addiction. 2008;103(10):1648-57. 32. Tobin KE, Sherman SG, Beilenson P, Welsh C, Latkin CA. Evaluation of the Staying Alive programme: Training injection drug users to properly administer naloxone and save lives. International Journal of Drug Policy. 2009;20(2):131-6. 33. Wagner KD, Valente TW, Casanova M, Partovi SM, Mendenhall BM, Hundley JH, Gonzalez M, Unger JB. Evaluation of an overdose prevention and response training programme for injection drug users in the Skid Row area of Los Angeles, CA. International Journal of Drug Policy. 2010;21(3):186-93. 34. Walley AY, Doe-Simkins M, Quinn E, Pierce C, Xuan Z, Ozonoff A. Opioid overdose prevention with intranasal naloxone among people who take methadone. Journal of Substance Abuse Treatment. 2013;44(2):241-7. 35. Walley AY, Xuan ZM, Hackman HH, Quinn, E, Doe-SimkinsM, Sorensen-Alawad, A, Ruiz, S, Ozonoff, A. Opioid overdose rates and implementation of overdose education and nasal naloxone distribution in Massachusetts: interrupted time series analysis. Bmj. 2013;346(f174). 36. Williams AV, Marsden J, Strang J. Training family members to manage heroin overdose and administer naloxone: Randomized trial of effects on knowledge and attitudes. Addiction. 2014;109(2):250-9. 37. Yokell MA, Green T. C, Bowman S, McKenzie M, Rich J. D. Opioid overdose prevention and naloxone distribution in Rhode Island. Medicine and health, Rhode Island. 2011;94(8):240-2. 38. Dahlem CHG, King L, Anderson G, Marr A, Waddell JE, Scalera M. Beyond rescue: Implementation and evaluation of revised naloxone training for law enforcement officers. Public Health Nursing. 2017; 34(6): 516-521. 39. Dudley L. S, Konomos D, Robbins V, Qiu L, Bauter R, Merlin MA. (2017). Opioid crisis at the Jersey Shore-special report. Journal of Public Health. 2017; 1-6. 40. Fisher R, O’Donnell D, Ray B, Rusyniak D. Police Officers Can Safely and Effectively Administer Intranasal Naloxone. Prehospital Emergency Care. 2016; 20(6):675-680. 41. Heavey SC, Delmerico AM, Burstein G, Moore C, Wieczorek WF, Collins RL, Chang Y, Homish GG. Descriptive epidemiology for community-wide naloxone administration by police officers and firefighters responding to opioid overdose. Journal of Community Health. 2018; 43(2):304-311. 42. Rando J, Broering D; Olson JE, Marco C, Evans SB. Intranasal naloxone administration by police first responders is associated with decreased opioid overdose deaths. American Journal of Emergency Medicine. 2015; 33(9): 1201-1204. http://cochranelibrary-wiley.com/o/:10.1016/j.ajem.2015.05.022 43. Simmons J, Rajan S, Goldsamt L, Elliott L. Implementation of online opioid overdose prevention, recognition and response trainings for professional first responders: Year 1 survey results. Drug & Alcohol Dependence. 2016; 169: 1-4. 44. Wagner KD, Bovet LJ, Haynes B, Joshua A, Davidson PJ. Training law enforcement to respond to opioid overdose with naloxone: Impact on knowledge, attitudes, and interactions with community members. Drug & Alcohol Dependence. 2016;165: 22-28.   **(Group 2: 2018-2022, N=9)**   1. Lintzeris N, Monds LA, Bravo M, Read P, Harrod ME, Gilliver R, Wood W, Nielsen S, Dietze PM, Lenton S, Shanahan M(11), Jauncey M, Jefferies M, Hazelwood S, Dunlop AJ, Greenaway M, Haber P, Ezard N, Malcom A. Designing, implementing and evaluating the overdose response with take-home naloxone model of care: An evaluation of client outcomes and perspectives. Drug Alcohol Rev 2020;39(1):55-65. 2. Buresh M, Gicquelais RE, Astemborski J, Kirk GD, Mehta SH, Genberg BL. Fatal overdose prevention and experience with naloxone: A cross-sectional study from a community-based cohort of people who inject drugs in Baltimore, Maryland. PLoS One 2020;15(3):e0230127. 3. Jones JD, Campbell AN, Brandt L, Metz VE, Martinez S, Wall M, Corbeil T, Andrews H, Castillo F, Neale J, Strang J, Ross S, Comer SD. A randomized clinical trial of the effects of brief versus extended opioid overdose education on naloxone utilization outcomes by individuals with opioid use disorder. Drug Alcohol Depend 2022;237:109505. 4. Troberg K, Isendahl P, Blomé MA, Dahlman D, Håkansson A. Characteristics of and Experience Among People Who Use Take-Home Naloxone in Skåne County, Sweden. Front Public Health. 2022 Mar 10;10:811001. doi: 10.3389/fpubh.2022.811001. PMID: 35359781; PMCID: PMC8960176. 5. Williams S, King T, Papamihail K, Buxton JA. The implementation and role of a staff naloxone program for non-profit community-based sites in British Columbia : A descriptive study. PLOS ONE 2021;16(5): e0251112. https://doi.org/10.1371/journal.pone.0251112 6. Janssen A, Garove B, LaBond V. Naloxone administration by nonmedical providers- a descriptive study of County sheriff department training. Subst Abuse Treat Prev Policy 2020;15(1):86. doi: 10.1186/s13011-020-00327-w. 7. Yang C, Favaro J, Meacham MC. NEXT Harm Reduction: An Online, Mail-Based Naloxone Distribution and Harm-Reduction Program. Am J Public Health. 2021 Apr;111(4):667-671. 8. Thylstrup B, Hesse M, Jørgensen M, Thiesen H. One opioid user saving another: the first study of an opioid overdose-reversal and naloxone distribution program addressing hard-to-reach drug scenes in Denmark. Harm Reduct J 2019;16(1):66. 9. Naumann RB, Durrance CP, Ranapurwala SI, Austin AE, Proescholdbell S, Childs R, Marshall SW, Kansagra S, Shanahan ME. Impact of a community-based naloxone distribution program on opioid overdose death rates. Drug Alcohol Depend 2019;204:107536. |
| --- |

**[Table S3. Summary of Quality of Execution (QoE) items in 41 studies with individual-level data, Group 1: 2003-2018](#OLE_LINK3)**

| **QoE item** | **Response** | **No. studies cmty (n=34)** | **No. studies law enforcement(n=7)** | **Total (n=41)** |
| --- | --- | --- | --- | --- |
| **1. Clearly specified target pop (for naloxone distribution)** | Yes | 29 | 7 | 36 |
|  | No | 5 |  | 5 |
| **1a. Specified drug using characteristics of target population** | Yes | 16 |  | 16 |
|  | No | 16 |  | 16 |
|  | Not Applicable (target pop not drug users) | 2 | 7 | 9 |
| **2. Thoroughness of OEND program description** | Very thorough | 8 | 1 | 9 |
|  | Thorough | 9 | 5 | 14 |
|  | Not very thorough | 17 |  | 17 |
|  | Not applicable |  | 1 | 1 |
| **3. Completeness of naloxone preparation description** | Complete | 16 | 2 | 18 |
|  | Partly complete | 9 | 3 | 12 |
|  | Not very complete | 9 | 1 | 10 |
|  | Not applicable |  | 1 | 1 |
| **4. Clarity of study design description** | Clear | 27 | 5 | 32 |
|  | Partly clear | 3 | 1 | 4 |
|  | Not clear | 4 | 1 | 5 |
| **5. Independence of evaluator-program relationship** | Strictly independent | 5 | 1 | 6 |
|  | Somewhat independent | 5 | 3 | 8 |
|  | Not very independent | 9 | 1 | 10 |
|  | Not at all independent | 6 | 1 | 7 |
|  | Relationship not described/can’t tell | 9 | 1 | 10 |
| **6. Description/characterization of study participant sampling** | Sampling described, bias addressed | 5 | 1 | 6 |
|  | Sampling described, there was no selection | 3 | 2 | 5 |
|  | Sampling described, bias not addressed | 16 | 2 | 18 |
|  | Sampling not described | 10 | 2 | 12 |
| **7. Clarity of outcomes specification (methods)** | Clear | 24 | 7 | 31 |
|  | Partly clear | 1 |  | 1 |
|  | Not clear | 9 |  | 9 |
| **8. Clarity of reporting of overdose outcomes** | Clear | 17 | 3 | 20 |
|  | Partly clear | 8 | 3 | 11 |
|  | Not clear | 9 | 1 | 10 |
| **8a. Completeness of reporting of overdose outcomes** | Complete | 12 | 3 | 15 |
|  | Partly complete | 10 | 3 | 13 |
|  | Not complete | 12 | 1 | 13 |
| **9. Presentation of findings** | Clear | 19 | 3 | 22 |
|  | Partly clear | 6 | 3 | 9 |
|  | Not clear | 9 | 1 | 10 |
| **10. Authors’ consideration of study limitations** | Considered and addressed appropriately | 6 |  | 6 |
|  | Considered, but not addressed or mitigated | 20 | 7 | 27 |
|  | Not well considered or addressed | 8 |  | 8 |

| **[Table S4. Intervention characteristics of community-based naloxone distribution programs in 41 studies with individual-level outcome data, by target population, Group 1: 2003-2018](#OLE_LINK4)** | | | | | | | | |
| --- | --- | --- | --- | --- | --- | --- | --- | --- |
| **Author (Year)** | **location** | **program name** | **intervention start year** | **venue** | **training duration** | **naloxone administration type** | **nalox dose** | **nalox doses/kit** |
| **PWUD** |  |  |  |  |  |  |  |  |
| **Dettmer (2001)** | Berlin, Germany and Jersey, Channel Islands, England | *The Berlin Project; The Jersey Project* | 1998 | mobile vans, ambulance, local drug services | NR | inject_1dosevial,  1 minijet filled with naloxone (Jersey) | 0.4 mg (Berlin), 0.8 mg (Jersey) | 2 |
| **Seal (2005)** | San Francisco CA | *Overdose Prevention and Management Program* | 2001 | community-based field sites | 8 hrs | prefilled injection cartridges of naloxone with injection devices | 0.4 mg | 2 |
| **Maxwell (2006)** | Chicago IL | *Not specified - Chicago Recovery Alliance* | 2001 | mobile vans, storefronts, areas of cell phone, pager access | 90 mins | inject_10dosevial | 0.4 mg | one 10mL vial |
| **Galea (2006)** | New York City NY | *Overdose Prevention and Reversal Program* | 2004 | ssp | approx 1 hr | inject_prefilled | 0.4 mg | 2 |
| **Heller (2007)** | New York City NY | *NYC DOHMH Initiative* | 2004 | ssp | 9-31 mins | inject_prefilled | 0.4 mg | 2 |
| **Piper (2008)** | New York City NY | *Skills and Knowledge on Overdose Prevention (SKOOP)* | NR | ssp | 10-30 mins | inject_prefilled | 1.0 mg | 2 |
| **Strang (2008)** | England | NR | NR | SUTP, criminal justice intervention programs | NR | Injection (unspecified) | NR | NR |
| **Tobin (2009)** | Baltimore MD | *Staying Alive programme (SA)* | 2004 | ssp | 1 hr | inject_10dosevial | 0.4 mg | one 10mL vial |
| **Doe-Simkins (2009)** | Boston MA | *NR* | 2006 | ssp | 15 mins | nasal_prefilled | 2.0 mg | 2 |
| **Author (Year)** | **location** | **program name** | **intervention start year** | **venue** | **training duration** | **naloxone administration type** | **nalox dose** | **nalox doses/kit** |
| **Enteen (2010)** | San Francisco CA | *Drug Overdose Prevention and Education (DOPE) Project* | 2003 | ssp, SUTP, cmty cntr | 10-30 mins | inject_1dosevial | 0.4 mg | 2 |
| **Wagner (2010)** | Los Angeles CA | *Homeless Health Care LA Center for Harm Reduction overdose prevention and response training program* | 2006 | ssp | 1 hr | inject_prefilled | 0.4 mg | 2 |
| **McAuley (2010)** | Lanarkshire Scotland | *Lanarkshire Naxone Pilot* | NR | NR | 4 hrs | inject_prefilled | 0.4 mg | 1 |
| **Bennett (2011)** | Pittsburgh PA | *Prevention Point Pittsburgh Overdose Prevention Program* | 2005 | ssp | 25 mins | **injected** | NR | NR |
| **Yokell (2011)** | RI | *Preventing Overdose and Naloxone Intervention (PONI)* | 2006 | NR | NR | inject_10dosevial | NR | one 10mL vial |
| **Dong (2012)** | Edmonton Canada | *Streetworks naloxone program* | 2005 | ssp | 30-45 mins | Injection (unspecified) | NR | NR |
| **Bennett (2012)** | Wales | *Wales Take Home Naloxone Demonstration Project* | 2011 | community sites and four prisons | NR | **injected** | 0.4 mg | 1 |
| **Lankenau (2013)** | Los Angeles CA | *HHCLA and CGW Overdose Prevention Programs* | 2006 | shelter, HIV clinic | NR | Injection (unspecified) | NR | 2 |
| **Walley (2013a)** | MA | *Massachusetts Opioid Overdose Prevention Pilot program* | 2006 | ssp,SUTP,shelter,cmtycntr | 5-60 mins | nasal_prefilled | 2.0 mg | 2 |
| **Walley (2013b)** | MA | *Massachusetts OEND program* | 2006 | ssp, SUTP, cmty cntr, other cmty | 10-60 mins | nasal_prefilled | 2.0 mg | 2 |
| **Banjo (2014)** | British Colombia Canada | *British Columbia Take Home Naloxone (BCTHN) program* | 2012 | other cmty | NR | Injection (unspecified) | 1.0 mg | NR |
| **Doe-Simkins (2014)** | Boston and Cambridge MA | *MDPH OEND program* | 2006 | ssp,SUTP,shelter,cmtycntr | 5-60 mins | nasal_prefilled | 2.0 mg | 2 |
| **Author (Year)** | **location** | **program name** | **intervention start year** | **venue** | **training duration** | **naloxone administration type** | **nalox dose** | **nalox doses/kit** |
| **Rowe (2015)** | San Francisco CA | *Drug Overdose Prevention and Education (DOPE) Project* | 2003 | ssp, SUTP, re-entry programs, single room occupancy hotels | 5-10 mins | Injection (unspecified) Nasal (unspecified) | Injection (**0.4 mg**) Nasal (**2.0 mg**) | 2 |
| **Parmar (2016)** | England | *The NAL-oxone InVEstigation (N-ALIVE) pilot trial* | 2011 | 16 prisons | NR | inject_prefilled | 1.0 mg | 1 |
| **Leece (2016)** | Toronto Ontario Canada | *Prevention Overdose in Toronto (POINT)* | 2011 | other cmty | 20 mins | inject_1dosevial | 0.4 mg | 2 |
| **Madah-Amiri (2017)** | Oslo Bergen Norway | *Norwegian THN project* | 2014 | ssp, shelter, prison | NR | nasal_prefilled | 0.8 mg | **2½** |
| **Pijl (2017)** | Alberta Canada | *Naloxone Distribution program for newly released inmates* | 2015 | correctional facility | NR | inject_1dosevial | 0.4 mg | 2 |
| **Freeman (2017)** | Alberta Canada | *Alberta's Provincial THN program* | 06/2015-07/2016 | ssp,SUTP,cmtycntr | NR | injected | 0.4 mg | 2 |
| **Bagley (2018)** | MA | *naloxone for family members in MDPH OEND* | 2007 | cmtycntr, healthcare settings | 20 mins | Nasal (unspecified) | NR | NR |
| **Bell (2018)** | Pittsburgh PA | *Prevention Point Pittsburgh (PPP)* | 2005 | ssp | NR | inject_1dosevial | 0.4 mg | 2 |
| **Katzman (2018)** | Albuquerque NM | *University of New Mexico Addiction and Substance Abuse Program (UNMASAP)* | 2016 | SUTP | NR | inject_auto | 0.4 mg | 2 |
| **FAMILY, OTHER** | |  |  |  |  |  |  |  |
| **Walley (2013b)** | MA | *Massachusetts Opioid Overdose Prevention Pilot program* | 2006 | ssp,SUTP,shelter,cmtycntr | NR | nasal_prefilled | 2.0 mg | 2 |
| **Williams (2014)** | London, Kent, Herefordshire, England | *Training familiy members to manage OD & administer naloxone* | 2009 | ssp,SUTP, family member support groups | 60 mins | inject_prefilled | 2.0 mg | 1 |
| **Author (Year)** | **location** | **program name** | **intervention start year** | **venue** | **training duration** | **naloxone administration type** | **nalox dose** | **nalox doses/kit** |
| **Bagley (2015)** | MA | *Learn to Cope OEN through MA Dept Public Health* | 2011 | cmtycntr | 20 mins | Nasal (unspecified) | NR | NR |
| **Lewis (2016)** | Baltimore City MD and 10 Maryland counties | *Baltimore Student Harm Reduction Coalition (BSHRC) OEND pram* | 2014 | cmtycntr,health orgs | NR | inject_1dosevial | 0.4 mg | 2 |
| **Bagley (2018)** | MA | *naloxone for family members in MDPH OEND* | 2007 | cmtycntr, healthcare settings | 20 mins | Nasal (unspecified) | NR | NR |
| **Huxley-Reicher (2017)** | Rikers Island NY | *Rikers Island Central Visiting Center Naloxone Pram* | 2014 | Rikers Island Central Visiting Center | 2-5 mins | nasal_prefilled | 1.0 mg | 2 |
| **LAW ENFORCEMENT PERSONNEL** | | |  |  |  |  |  |  |
| **Rando (2015)** | Lorain County OH | police officer naloxone prescription pram | 2013 |  | 2 hrs | Nasal (unspecified) | 2.0 mg | NR |
| **Wagner (2016)** | San Diego CA | LEO naloxone pram | 2014 |  | 30 mins | nasal_prefilled | 2.0 mg | 2 |
| **Fisher (2016)** | IN | Police Officer Naloxone Distribution Pram | 2014 |  | 30 mins | nasal_vial | 2.0 mg | 1 |
| **Simmons (2016)** | PA | online training of police | 2014 |  | 45 mins | Nasal (unspecified),  prefilled,inject_auto | NR | NR |
| **Dudley (2017)** | Monmouth and Ocean Counties NJ | NR | 2014 |  | NR | Nasal (unspecified) | NR | NR |
| **Dahlem (2017)** | Washtenaw County MI | Ann Arbor Naloxone Training for Law Enforcement Officers | 2015 |  | 45-60 mins | nasal_vial | NR | 1 |
| **Heavey (2018)** | Erie County NY | Erie County Police and Fire Fighters Naloxone Administration Pram | 2014 |  | NR | nasal_prefilled | NR | 2 |

NR = not reported; ssp = syringe services program; SUTP = Substance use treatment program;

| **[Table S5. Study characteristics and selected outcomes of community-based naloxone distribution programs by population subgroup: Group 1: (2003-2018) (N=44)](#OLE_LINK5)** | | | | | | | | | | | |  |
| --- | --- | --- | --- | --- | --- | --- | --- | --- | --- | --- | --- | --- |
| **Author (Year)** | **location** | **study location** | **study dates** | **study design** | **type follow up** | **#ppts recd nlx** | **#ppts in fu** | **#ODs nlx used** | **# survivals** | **# deaths** | **survival proportion (%)** | |
| **PWUD** |  |  |  |  |  |  |  |  |  |  |  | |
| Dettmer (2001) | Europe | Berlin, Germany and Jersey, England | NR | prospective cohort | passive | 124 (Berlin) 101 (Jersey) | 40 (Berlin) 5 (Jersey) | 29 (Berlin) 5 (Jersey) | 29 (Berlin) 5 (Jersey) | 0 | 100% | |
| Seal (2005) | US | San Francisco CA | 08/2001-01/2002 | prospective cohort | scheduled: 6mths | 24 | 24 | 15 | 15 | 0 | 100.0% | |
| Maxwell (2006) | US | Chicago IL | Not specified | prospective cohort | passive | ≥3,500 | ≤320 | 320 | 319 | 1 | 99.7% | |
| Galea (2006) | US | New York City NY | 06/2004-01/2005 | prospective cohort | scheduled: 3mths and passive | 25 | 22 | 10 | 10 | 0 | 100.0% | |
| Heller (2007) | US | New York City NY | NR | prospective cohort | NR | 1,800 | ≤162 | 162 | 162 | 0 | 100.0% | |
| Piper (2008) | US | New York City NY | 03/2005-12/2005 | prospective cohort | passive | 122 | NA | 82 | 68 | 0 | 82.9% | |
| Strang (2008) | Europe | England | NR | prospective cohort | scheduled: 3mths | 239 | 186 | 10 | 10 | 0 | 100.0% | |
| Tobin (2009) | US | Baltimore MD | 10/2004-04/2005 | prospective cohort | scheduled: 6mths | 250 | 85 | 22 | 22 | 0 | 100.0% | |
| Doe-Simkins (2009) | US | Boston MA | 09/2006-12/2007 | prospective cohort | passive | 385 | 279 | 74 | 74 | 0 | 100.0% | |
| Enteen (2010) | US | San Francisco CA | 09/2003-12/2009 | prospective cohort | passive | 1,942 | 470 | 399 | 357 | 6 | 89.5% | |
| Wagner (2010) | US | Los Angeles CA | 09/2006-01/2008 | prospective cohort | scheduled: 3mths | 66 | 47 | 28 | 26 | 4 | 92.9% | |
| McAuley (2010) | Europe | Lanarkshire Scotland | NR | prospective cohort | scheduled: 2 and 6 mths | 19 | 17 | 2 | 2 | 0 | 100.0% | |
| Bennett (2011) | US | Pittsburgh PA | 07/2005-12/2008 | prospective cohort | passive | 426 | 141 | 249 | 239 | 2 | 96.0% | |
| Yokell (2011) | US | RI | 2006 | prospective cohort | scheduled: 3mths and passive | 120 | 10 | 5 | 5 | 0 | 100.0% | |
| **Author (Year)** | **location** | **study location** | **study dates** | **study design** | **type follow up** | **#ppts recd nlx** | **#ppts in fu** | **#ODs nlx used** | **# survivals** | **# deaths** | **survival proportion (%)** | |
| Dong (2012) | Canada | Edmonton | 07/2006-07/2007 | prospective cohort | scheduled: 12mths | 50 | 15 | 9 | 9 | 0 | 100.0% | |
| Bennett (2012) | Europe | Wales | NR | prospective cohort, pre-post with comparison group | passive | 525 | 28 | 28 | 27 | 1 | 96.4% | |
| Lankenau (2013) | US | Los Angeles CA | 2010-2011 | retrospective cohort | NA | 30 | NA | 15 | 15 | 0 | 96.7% | |
| Walley, Doe-Simkins (2013a) | US | MA | 09/2008-12/2010 | prospective cohort | passive | 1,553 | 62 | 92 | 92 | 0 | 100.0% | |
| Walley, Xuan (2013b) | US | MA | 09/2006-12/2009 | retro cohort and interrupted time series | NA | 4857 | ≤545 | 545 | NR | NR |  | |
| Banjo (2014) | Europe | British Colombia Canada | 11/2012 to 06/2013 | cross-sectional | NA | 692 | ≤126 | 83 | 83 | NR | 100.0% | |
| Doe-Simkins (2014) | US | Boston and Cambridge MA | 09/2006-12/2010 | retrospective cohort | NA | 4,926 | 325 | 508 | 295 | NR | 58.1% | |
| Rowe (2015) | US | San Francisco CA | 01/2010-12/2013 | prospective cohort | passive | 2,500 | 613 | 702 | 658 | 10 | 93.7% | |
| Parmar (2016) | Europe | 16 prisons in England | 05/2012 - 12/2014 | randomized | passive | 659 | 112 | 23 | 17 | NR | 73.9% | |
| Leece (2016) | Canada | Toronto Ontario | 08/2011-8/2013 | prospective cohort | passive | 662 | 67 | 98 | 95 | 1 | 96.9% | |
| Madah-Amiri (2017) | Europe | Oslo Bergen Norway | 06/2014-12/2015 | prospective cohort | passive | 1,322 | 734 | 277 | 265 | 0 | 95.7% | |
| Pijl (2017) | Canada | Alberta | 10/2015-03/2016 | prospective cohort | passive | 54 | 4 | 4 | 4 | 0 | 100.0% | |
| Freeman (2017) | Canada | Alberta | 07/2015-07/2016 | cross-sectional | NA | 2,910 | 472 | 472 | 472 | NR | 100.0% | |
| Bagley (2018) | US | MA | 2008-2015 | retrospective cohort | NA | 4,679 | ≤688 | 688 | 666 | 12 | 96.8% | |
| **Author (Year)** | **location** | **study location** | **study dates** | **study design** | **type follow up** | **#ppts recd nlx** | **#ppts in fu** | **#ODs nlx used** | **# survivals** | **# deaths** | **survival proportion (%)** | |
| Bell (2018) | US | Pittsburgh PA | 01/2013-12/2016 | retrospective cohort | NA | 1,072 | NA | 1,072 | 1,072 | NR | 100.0% | |
| Katzman (2018) | US | Albuquerque NM | 04/2016-07/2016 | prospective cohort | scheduled: 3mths and passive | 244 | 215 | 38 | 38 | 0 | 100.0% | |
| Rowe (2016) | US | San Francisco CA | 01/2010-12/2012 | ecologic | NA |  |  |  |  |  |  | |
| Rowe (2019) | US | San Francisco CA | 01/2014-12/2015 | ecologic | NA |  |  |  |  |  |  | |
| **FAMILY, OTHER** | |  |  |  |  |  |  |  |  |  |  | |
| Walley (2013b) | US | MA | 09/2006-12/2009 | retrospective cohort and interrupted time series | NA | 905 | NA | 41 | 20 | 0 | 100.0% | |
| Williams (2014) | Europe | London Kent Herefordshire England | 2009-2010 | randomized | scheduled: 3mths | 84 | 69 | 2 | 2 | 0 | 100.0% | |
| Bagley (2015) | US | MA | 07/2013-09/2013 | cross-sectional | NA | 99 | NA | 5 | 5 | 0 | 100.0% | |
| Lewis (2016) | US | Baltimore City MD and 10 Maryland counties | 04/2014-11/2014 | prospective cohort | scheduled: 8-12mths | 250 | 35 | 3 | 3 | 0 | 100.0% | |
| Bagley (2018) | US | MA | 2008-2015 | retrospective cohort | NA | 40,801 | ≤4373 | 4,373 | 4,229 |  | 96.7% | |
| Huxley-Reicher (2017) | US | Rikers Island NY | 08/2015 (five consecutive days) | prospective cohort | scheduled: 6mths | 382 | 226 | 55 | 50 | 3 | 90.9% | |
| **LAW ENFORCEMENT PERSONNEL** | | |  |  |  |  |  |  |  |  |  | |
| Rando (2015) | US | Lorain County OH | 01/2011-10/2014 | prospective cohort | NR | NR |  | 67 | 52 | 7 | 77.6% | |
| Simmons (2016) | US | PA | 01/2015-10/2015 | prospective cohort | NR | 207 |  | 88 | 69 | NR | 78.4% | |
| Wagner (2016) | US | San Diego CA | 07/2014-11/2014 | prospective cohort | NA | 83 |  | 11 | 9 | 2 | 81.8% | |
| Fisher (2016) | US | IN | 04/2014-09/2015 | retrospective cohort | NA | 900 |  | 126 | 104 | 3 | 82.5% | |
| **Author (Year)** | **location** | **study location** | **study dates** | **study design** | **type follow up** | **#ppts recd nlx** | **#ppts in fu** | **#ODs nlx used** | **# survivals** | **# deaths** | **survival proportion (%)** | |
| Dahlem (2017) | US | Washtenaw County MI | 08/2015-08/2016 | prospective cohort | NR | 109 |  | 32 | 31 | 1 | 96.9% | |
| Dudley (2017) | US | Monmouth and Ocean Counties NJ | 01/2016-04/2016 | retrospective cohort | NA | NR |  | 312 | 282 | 30 | 90.4% | |
| Heavey (2018) | US | Erie County NY | 07/2014-07/2016 | prospective cohort | NR | NR |  | 800 | 653 | 50 | 81.6% | |

#ppts recd nlx = number of OEND program participants who were trained and received naloxone kits

#ppts in fu = number of OEND program participants who returned in follow-up

#ODs nlx used = number of overdoses for which naloxone was used/administered

**[Additional findings](#OLE_LINK6)**

1. Changes over **time (years)**:

Changes in OEND program characteristics over time reflected the evolution of innovations in program practices and policies. Early programs studied distributed injectable naloxone and sterile syringes to people who injected drugs; later programs distributed nasal naloxone and began recruiting family members and other community members and, still later, public safety personnel.

| **[Table S6. Characteristics of studies of naloxone distribution programs: Secular trends overtime (years (N=41)](#OLE_LINK7)** | | | | | | | | | | | | | | |
| --- | --- | --- | --- | --- | --- | --- | --- | --- | --- | --- | --- | --- | --- | --- |
| **Study midpoint** |  | Population served* | | | |  |  |  | Naloxone used | | | |  | Training duration  ≥ 1 hr (%) |
|  |  | people who used drugs | family members | police | Total studies |  |  |  |  |  |  | Total studies |  |  |
|  |  |  |  |  |  |  |  |  | inject | nasal |  |  |  |  |
| **1999-2004** |  | 4 | 0 | 0 | 4 |  | **1999-2004** |  | 4 | 0 |  | 4 |  | 100% |
| **2005-2007** |  | 10 | 0 | 0 | 10 |  | **2005-2007** |  | 9 | 1 |  | 10 |  | 25% |
| **2008-2010*** |  | 6 | 2 | 0 | 7* |  | **2008-2010** |  | 4 | 3 |  | 7 |  | 40% |
| **2011-2013*** |  | 5 | 2 | 1 | 7* |  | **2011-2013*** |  | 4 | 4 |  | 7* |  | 0% |
| **2014-2016** |  | 5 | 2 | 6 | 13 |  | **2014-2016*** |  | 6 | 8 |  | 13* |  | 0% |
| **Total** |  | 30 | 6 | 7 | 41* |  | **Total** |  | 27 | 16 |  | 41* |  |  |
|  |  | *2 studies included both PWUD and family members | | | |  | *2 studies used both nasal and injected naloxone | | | | | | | |
| Studies were grouped by study midpoint into six three-year intervals. The first two intervals (1999-2004) were combined because they contained only 4 studies. | | | | | | | | | | | | | | |

- The proportion of studies using nasal naloxone trended upward over time:       0% ▢ 10% ▢ 43% ▢ 57% ▢ 62%.
- The proportion of studies of interventions serving families increased from        0% ▢   0% ▢ 29% ▢ 29% ▢ 15%.
- The proportion of studies of interventions with public safety increased from    0% ▢   0% ▢   0% ▢ 14% ▢ 46%.

Training **duration** declined over time, as programs switched to nasal naloxone. (Studies of programs using nasal naloxone reported training participants for shorter durations. Half of 12 programs distributing injectable naloxone trained participants for ≥ 1 hour; none of 10 studies distributing nasal naloxone did.)

- The proportion of studies with ≥ 1 hour of training declined over time:               100% ▢25% ▢ 40% ▢ 0% ▢ 0%.

There were no trends over time in study size, program location, follow-up method, follow-up, kit usage, or survival proportion.*

1. Findings by **location**:

- Studies in the **U.S.** were more than 4 times **larger** than those elsewhere (mean no. ppts receiving naloxone/study, **2,514** vs. **572**). They were also **more numerous** than those elsewhere. We found **31 studies** conducted in the United States that met our inclusion criteria, but only **13** in other countries: **6** in the **UK**, **5** in **Canada**, **2** in **other European countries**, and **none** in **Australia**.
- **Follow-up** was greater outside the US (self-weighted mean, **24.5**% vs. **11.9**%) — even though the proportion of studies that used prospectively scheduled (vs. passive) follow-up was slightly **lower** in non-US studies (**30.8**% vs. **36.4**%).
- There were no apparent difference in **survival proportion** or other parameters.

1. Findings by **follow-up method**:

- Studies with prospectively scheduled follow-up, unsurprisingly, had considerably **greater follow-up** than those with passive follow-up only (self-weighted mean, **54.2%** vs. **12.1%**).
- However, studies with passive follow-up were **forty times larger**, and **survivals** per study were also **forty times** those with scheduled follow-up.

These observations reflect the tradeoff between the two types of studies. Studies that reviewed program records for all program participants examined larger, more representative samples, at the cost of having lower follow-up. Studies that enrolled participants prospectively selected smaller, less representative subsets of people willing to enroll in a study and provide follow-up. Both types of studies provide useful information.

1. Findings by **route of naloxone administration:**

- Studies of nasal naloxone were **nearly 10 times as large** as those of injected naloxone (mean no. ppts receiving naloxone/study, **5,021** vs. **615**).
- Nasal naloxone **usage per kit distributed** was lower (self-weighted mean, **9.6**% vs. **16.3**%).
- Nonetheless, because of the larger number of participants, the number of naloxone uses per study was greater in the studies of nasal naloxone than injected naloxone (mean, **5,968** vs. **3,185**).
- **Survival** was similar in studies of nasal (**97.9%** [95%CI: 96.2-98.8]) and injected (**97.7%** [95%CI: 94.1-97.9], p-value=0.212) naloxone in mixed effects models.
- Nonetheless, because of the larger study sizes, the **number of survivals per study** was greater in the studies of nasal naloxone than injected naloxone (mean, **5,161** vs. **3,106**).

1. Findings by **naloxone dose** in studies of injected naloxone:

- Among studies using injected naloxone, those using 0.4 mg doses were more numerous (16 vs. 5) than studies using higher doses, and larger (mean no. of participants receiving naloxone, **842** vs. **332**), and, despite lower follow-up (self-weighted mean, **16.2**% vs. **20.3**%) had higher usage (self-weighted mean, **16.8**% vs. **9.2**% of kits distributed). Survival in studies of programs using 0.4 mg doses (**98.2%** [95%CI: 95.8-99.2]) and those using higher doses (**97.0%** [95%CI: 94.1-98.5]) was similar (p-value=0.36). The higher doses were 0.8 mg in one study, 1.0 in three, and 2.0 in one.

The appropriate dose for programs to use is an important issue. From the start, the first priority of EMTs with an unresponsive patient has always been to rapidly reverse the unresponsiveness if opioid overdose was the cause and rapidly determine if it was not. For those reasons, they used 2.0 mg, although it usually precipitated severe withdrawal symptoms that were startling and painful for the patient. The priorities of people using drugs (usually earlier to the scene if not already there) were to use the minimum dose necessary to reverse the overdose, repeating the dose as needed to more gently arouse their companion or peer to safety.

*Note: For survival proportions, summary estimates of data pooled from multiple studies were calculated using random effects models in meta-analysis. For other outcomes, self-weighted means are shown. Dividing total numerators from all studies by total denominators from all studies results in a summary mean value that is weighted by the denominator in each study.

**References**

Horowitz Z. Subcutaneous naloxone: a less rude awakening? Acad Emerg Med 1998 Apr;5(4):283-5. doi: 10.1111/j.1553-2712.1998.tb02705.x. PMID: 9562188.

Payne ER, Stancliff S, Rowe K, Christie JA, Dailey MW. Comparison of Administration of 8-Milligram and 4-Milligram Intranasal Naloxone by Law Enforcement During Response to Suspected Opioid Overdose — New York, March 2022–August 2023. MMWR 2024;73:110–3. doi: <http://dx.doi.org/10.15585/mmwr.mm7305a4>.

Compassionate Overdose Response Summit and Naloxone Dosing Meeting. March 18-19, 2024. <https://www.healthmanagement.com/insights/webinars/compassionate-overdose-response-summit-and-naloxone-dosing-meeting/>

1. Findings by **training duration**:

- Survival in studies that provided participants 30 minutes or less of training in overdose prevention and response (**97.8%** [95%CI: 96.8-98.5]), 31-60 minutes (**96.9%** [95%CI: 93.5-98.6]), or 61 minutes or more (**96.1%** [95%CI: 76.4-99.5]) were similar (p=0.65).
- However, study size was inversely related to duration of training (mean no. ppts receiving naloxone/study, **3,468** in studies with up to 60 minutes of training, **106** in those with one hour, and **22** in those with 4-8 hours of training).

**Figure S1A:** Funnel plot to check for the existence of publication bias


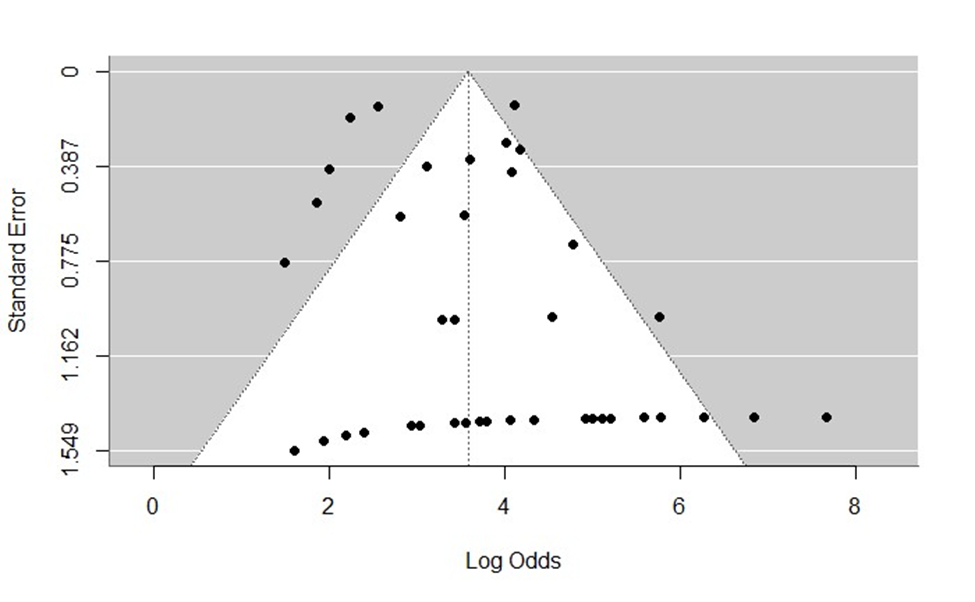


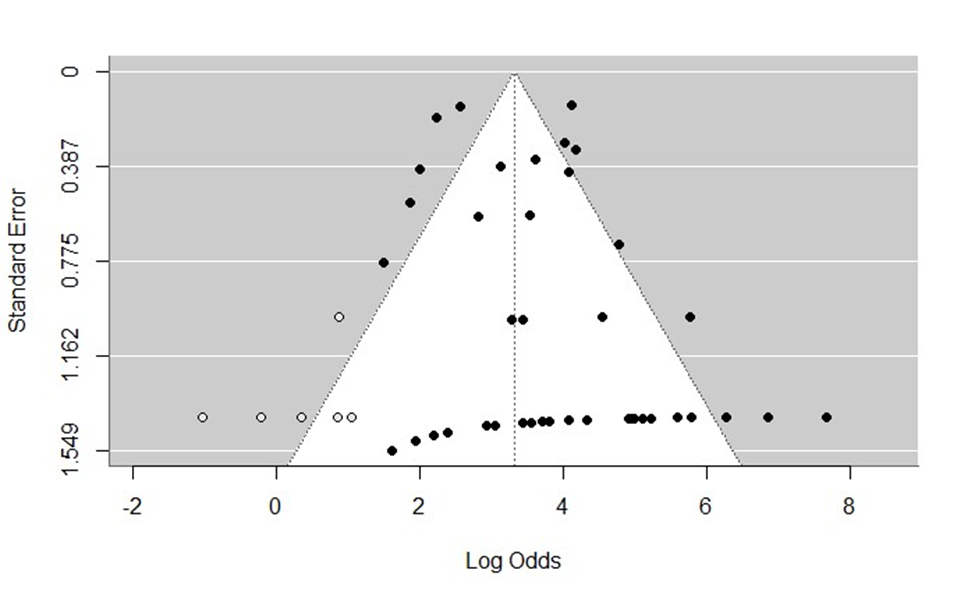
**Figure S1B:** Funnel plot with trim-and-fill to estimate potential impact of imputed “missing” studies

**Figure S1:** The funnel plot (Figure S1A) indicates our study may be missing studies from the bottom left quadrant, which would be studies with lower survival and higher variance (Shi, 2019). The trim-and-fill method added in 6 imputed “missing” studies (plotted as white circles, Figure S1B). Adjusting overall summary estimates, no substantial impact of publication bias on our summary estimates was observed. The original estimate of survival was 97.3% (95% CI: 96.1-98.3); with adjustment using trim-and-fit methodology, it was 96.5% (95.0 - 97.6).

**Reference:** Shi L, Lin L. The trim-and-fill method for publication bias: practical guidelines and recommendations based on a large database of meta-analyses. Medicine (Baltimore) 2019;98(23):e15987. doi: 10.1097/MD.0000000000015987.
